# Supplementary material for: Partitioning to ordered membrane domains regulates the kinetics of secretory traffic
Source: eLife. 2024 Jun 5;12:RP89306. doi: 10.7554/eLife.89306 (PMC11152573; doi:10.7554/eLife.89306)
Supplement: Supplementary file 1. [file elife-89306-supp1.docx]

**Supplemental Information for:**

**Partitioning to ordered membrane domains regulates the kinetics of secretory traffic**

Ivan Castello-Serrano, Fred A. Heberle, Barbara Diaz-Rohrer, Rossana Ippolito, Carolyn R. Shurer, Pablo Lujan, Felix Campelo, Kandice R. Levental, Ilya Levental

**Table S1. Raft affinity (K_p,raft_) values for constructs used in this study.**

| **protein construct** | **K_p,raft_ (mean±SD)** |
| --- | --- |
| GPI | 1.64 ± 0.09 |
| LAT | 1.33 ± 0.04 |
| LAT-TMD | 1.35 ± 0.06 |
| allA8L-TMD | 1.14 ± 0.13 |
| LAT-allL | 0.60 ± 0.07 |
| allL-TMD | 0.53 ± 0.01 |
| TfR | 0.37 ± 0.06 |
| LAX | 0.60 ± 0.02 |
| LAX-TMD | 0.76 ± 0.26 |
| VSVG | 0.50 ± 0.12 |
